# Plasma glial fibrillary acidic protein and neurofilament light chain, but not tau, are biomarkers of sports-related mild traumatic brain injury

**DOI:** 10.1093/braincomms/fcaa137

**Published:** 2020-09-07

**Authors:** Etienne Laverse, Tong Guo, Karl Zimmerman, Martha S Foiani, Bharat Velani, Philip Morrow, Ademola Adejuwon, Richard Bamford, Natasha Underwood, Jonathan George, Daniel Brooke, Karen O’Brien, Matthew J Cross, Simon P T Kemp, Amanda J Heslegrave, John Hardy, David J Sharp, Henrik Zetterberg, Huw R Morris

**Affiliations:** Department of Clinical and Movement Neurosciences, UCL Institute of Neurology, Queen Square, UK; Department of Clinical and Movement Neurosciences, UCL Institute of Neurology, Queen Square, UK; Division of Brain Sciences, Department of Medicine, Imperial College London, London, UK; Department of Neurodegenerative Disease, UK Dementia Research Institute at UCL, UCL Institute of Neurology, University College London, London, UK; Department of Clinical and Movement Neurosciences, UCL Institute of Neurology, Queen Square, UK; Saracens Rugby Club, UK; Ealing Trailfinders Rugby Club, London, UK; Saracens Rugby Club, UK; Saracens Rugby Club, UK; Saracens Rugby Club, UK; Ealing Trailfinders Rugby Club, London, UK; London Scottish Rugby Club, UK; Premiership Rugby, Twickenham, London, UK; Rugby Football Union, Twickenham, London, UK; Faculty of Epidemiology and Population Health, London School of Hygiene and Tropical Medicine, London, UK; Department of Neurodegenerative Disease, UK Dementia Research Institute at UCL, UCL Institute of Neurology, University College London, London, UK; Department of Neurodegenerative Disease, UCL Queen Square Institute of Neurology, Queen Square, London WC1N 3BG, UK; Department of Neurodegenerative Disease, UK Dementia Research Institute at UCL, UCL Institute of Neurology, University College London, London, UK; Department of Neurodegenerative Disease, UCL Queen Square Institute of Neurology, Queen Square, London WC1N 3BG, UK; Reta Lila Weston Institute, UCL Queen Square Institute of Neurology, London WC1N 1PJ, UK; NIHR University College London Hospitals Biomedical Research Centre; Institute for Advanced Study, The Hong Kong University of Science and Technology, Hong Kong SAR, China; Division of Brain Sciences, Department of Medicine, Imperial College London, London, UK; Department of Neurodegenerative Disease, UK Dementia Research Institute at UCL, UCL Institute of Neurology, University College London, London, UK; Department of Neurodegenerative Disease, UCL Queen Square Institute of Neurology, Queen Square, London WC1N 3BG, UK; Department of Psychiatry and Neurochemistry, Institute of Neuroscience and Physiology, the Sahlgrenska Academy at the University of Gothenburg, Mölndal, Sweden; Clinical Neurochemistry Laboratory, Sahlgrenska University Hospital, Mölndal, Sweden; Department of Clinical and Movement Neurosciences, UCL Institute of Neurology, Queen Square, UK

**Keywords:** mild-TBI, concussion, biomarkers, NFL, GFAP

## Abstract

Mild traumatic brain injury is a relatively common event in contact sports and there is increasing interest in the long-term neurocognitive effects. The diagnosis largely relies on symptom reporting and there is a need for objective tools to aid diagnosis and prognosis. There are recent reports that blood biomarkers could potentially help triage patients with suspected injury and normal CT findings. We have measured plasma concentrations of glial and neuronal proteins and explored their potential in the assessment of mild traumatic brain injury in contact sport. We recruited a prospective cohort of active male rugby players, who had pre-season baseline plasma sampling. From this prospective cohort, we recruited 25 players diagnosed with mild traumatic brain injury. We sampled post-match rugby players without head injuries as post-match controls. We measured plasma neurofilament light chain, tau and glial fibrillary acidic protein levels using ultrasensitive single molecule array technology. The data were analysed at the group and individual player level. Plasma glial fibrillary acidic protein concentration was significantly increased 1-h post-injury in mild traumatic brain injury cases compared to the non-injured group (*P* = 0.017). Pairwise comparison also showed that glial fibrillary acidic protein levels were higher in players after a head injury in comparison to their pre-season levels at both 1-h and 3- to 10-day post-injury time points (*P* = 0.039 and 0.040, respectively). There was also an increase in neurofilament light chain concentration in brain injury cases compared to the pre-season levels within the same individual at both time points (*P* = 0.023 and 0.002, respectively). Tau was elevated in both the non-injured control group and the 1-h post-injury group compared to pre-season levels (*P* = 0.007 and 0.015, respectively). Furthermore, receiver operating characteristic analysis showed that glial fibrillary acidic protein and neurofilament light chain can separate head injury cases from control players. The highest diagnostic power was detected when biomarkers were combined in differentiating 1-h post-match control players from 1-h post-head injury players (area under curve 0.90, 95% confidence interval 0.79–1.00, *P* < 0.0002). The brain astrocytic marker glial fibrillary acidic protein is elevated in blood 1 h after mild traumatic brain injury and in combination with neurofilament light chain displayed the potential as a reliable biomarker for brain injury evaluation. Plasma total tau is elevated following competitive rugby with and without a head injury, perhaps related to peripheral nerve trauma and therefore total tau does not appear to be suitable as a blood biomarker.

## Teaser

Neuro-astrocytic proteins are potential blood markers of traumatic brain injury. We report significant increases in glial fibrillary acidic protein and neurofilament light chain in sports-related mild traumatic brain injury compared to post-match controls and demonstrate their potential as biomarkers; total-tau was increased in post-match controls and mild traumatic brain injury.

## Graphical Abstract

**Graphical Abstract:**
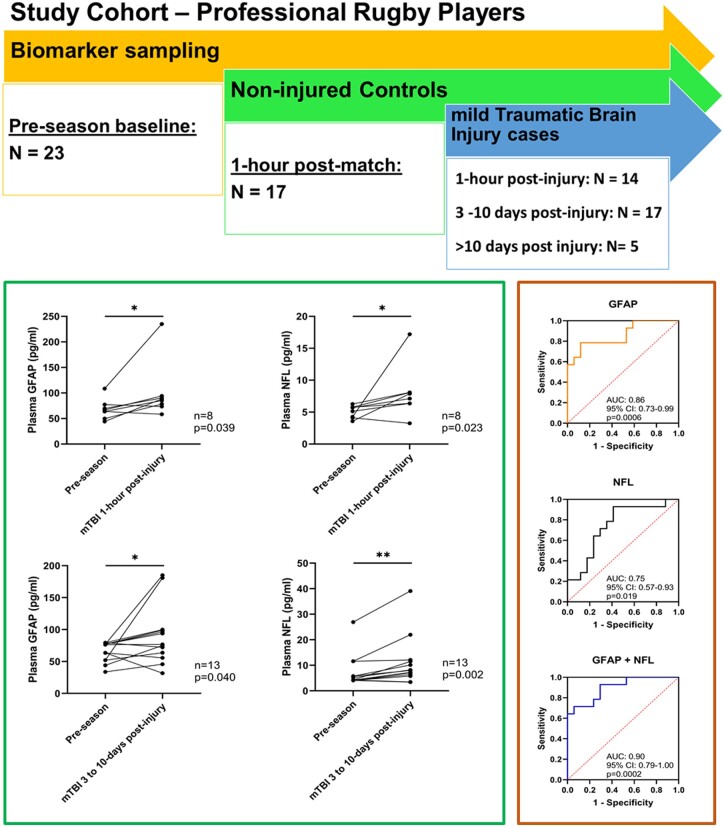

## Introduction

Concussion, also known as mild traumatic brain injury (mTBI), accounts for >80% of the estimated 1.4 million TBI cases seen in hospitals in the UK annually and affects 1.6–3.8 million individuals in the USA each year (Cancelliere *et al.*, 2014; Levin and Diaz-Arrastia, 2015). The syndrome comprises a constellation of symptoms following biomechanical injury to the brain. Angular or linear acceleration of the brain can result in neuronal shearing and dysfunction. Commonly reported symptoms include headache, visual disturbance, disorientation and memory impairment (Mayer *et al.*, 2017). The diagnosis and management of mTBI largely depends on detection of symptoms and signs (Dessy *et al.*, 2017). There is increasing recognition that repeated mTBIs may cause important neurocognitive sequelae and there is a clear need for objective tools to improve the current diagnostic approach (Ling *et al.*, 2017; Manley *et al.*, 2017; Adams *et al.*, 2018). Management guidelines in sports have been prepared largely from recognized consensus processes, and the need for more objective diagnostic tools, including fluid biomarkers, has been recognized as a priority (McCrory et al., 2017). At present, standard management in professional sports is that athletes with a suspected mTBI should leave the field of play. The key elements of the 10-min off-field World Rugby Head Injury Assessment 1 include review of game footage for presence of signs, memory and gait assessments and an mTBI symptom check (Raftery *et al.*, 2016; McCrory et al., 2017). However, athletes are known to under report symptoms and this can affect diagnosis (Meier *et al.*, 2015). Furthermore, the clinical evaluation remains limited as the relationship between extent of injury to the brain, particularly the microstructural changes that result from mTBI, and immediate clinical symptoms is uncertain. A variety of methods, including diffusion tensor imaging, have shown physiological changes that may persists longer than functional clinical recovery (Kamins *et al.*, 2017).

Neuronal and glial markers in blood and cerebrospinal fluid have been explored in previous studies. There is a reported correlation between biomarkers and head injury severity and symptom duration (Zetterberg *et al.*, 2009; Shahim *et al.*, 2014, 2017; Gill *et al.*, 2017; Bazarian *et al.*, 2018). Tau, which is primarily localized in axons is increased following sports-related mTBI (Neselius *et al.*, 2013; Battista *et al.*, 2016) and neurofilament light (NFL) levels, predominantly expressed in long myelinated axons, correlate with mTBI severity (Julien, 1999; Shahim *et al.*, 2017). There has been a recent report from a multicentre study of glial fibrillary acidic protein (GFAP), a biomarker of astrocyte injury, showing potential to have the discriminative ability to predict MRI abnormalities in TBI patients with normal CT findings (Yue *et al.*, 2019).

There is major interest in improving the acute management and long-term outlook for sports players with mTBI. Furthermore, intensive monitoring in rugby and a predictable rate of mTBI in contact sports makes this an ideal study group in which to explore the biology of mTBI. We hypothesized that fluid neuronal and glial biomarkers would be increased in mTBI and therefore help in establishing objective markers for the clinical evaluation of mTBI. This would allow for earlier diagnosis and an increased diagnostic yield, which, with effective management, may mitigate the long-term neurological sequelae.

## Materials and methods

Our protocol was approved by University College London (UCL) research ethics committee (7385/001). All participants provided written informed consent at study entry and baseline sampling, and consent was confirmed for additional samples. Active male players were recruited from Rugby Football Union and Rugby Football League in the UK in 2017–18. All players underwent standard mTBI-related assessments in the pre-season period, including the most recent version of the standardized Sport Concussion Assessment Tool 5, at the time of assessment. We followed the cohort prospectively during the active playing season and sampled players after a diagnosis of mTBI; non-head injured players were sampled as controls following a match.

### Assessment of mTBI

Experienced team physicians used internationally recognized consensus guidelines for the diagnosis of mTBI: Head Injury Assessment tools (Rugby Football Union players) and The Sport Concussion Assessment Tool. All head injuries met the Mayo Clinic classification of probable/mild-TBI, no injury involved prolonged loss of consciousness or amnesia (Malec *et al.*, 2007). We have further sub-categorized mTBI by (i) the presence or absence of permanent removal from play criteria (e.g. ataxia or tonic posturing), (ii) the number of reported symptoms, (iii) total number of symptomatic days and (iv) the duration the player remained in the return to play protocol.

### Fluid sampling

We obtained blood samples *via* venepuncture into EDTA (ethylenediaminetetraacetic acid) tubes. Samples were centrifuged within 20–60 min at the sports or training ground and the supernatants stored at −20°C and transferred to a −80°C within 2 weeks.

### Sampling time points

Pre-season, 1 h following suspected mTBI and 3–10 days following mTBI diagnosis, where available. Control subjects (with no mTBI) were sampled pre-season and 1 h after a routine match.

### Biochemical procedures

We measured NFL, total tau and GFAP concentrations in plasma using the Quanterix Simoa 4-Plex assay on the Simoa-HD1 following the manufacturer’s protocol (Quanterix Corp, Boston, MA, USA) (Rissin *et al.*, 2010). The assay was designed to also measure ubiquitin C-terminal hydrolase-L1 but this analyte showed intra-assay coefficients of variation above 20% and was deemed unreliable from a technical standpoint. Plasma samples were thawed on wet ice, centrifuged at 500 × g for 5 min at 4°C. Calibrators (neat) and samples (plasma: 1:4 dilution) were measured in duplicates. We analysed all samples at the same time using the same batch of reagents in a single plate. A four-parameter logistic curve fit data reduction method was used to generate a calibration curve. Two control samples of known concentration of the protein of interest (high-ctrl and low-ctrl) were included as quality control. Individual measurements fulfilling acceptance criteria were included in the analysis (accuracy = 80–120%, coefficient of variation of duplicate determination ≤20%). One pre-season sample failed tau measurement. The Single Molecule Array assay was conducted at the biomarker lab at the UK Dementia Research Institute at UCL.

### Statistical analysis

All statistical calculations were performed using GraphPad Prism (GraphPad, Inc., San Diego, CA and SPSS, Inc., Chicago, IL, USA) and SPSS ver.24 (IBM, Armonk, NY, USA). Normality for individual variables was tested by the Shapiro–Wilk test. Between-group comparisons were performed using the Kruskal–Wallis test with *post hoc* Dunn’s test. Wilcoxon matched-pairs signed rank test was used for within-player analysis. Receiver operating characteristic curve analysis was conducted to assess the diagnostic accuracy of each biomarker. All tests are two-tailed. Statistical significance was determined at *P* < 0.05 for all analyses.

### Data availability

Raw data were generated at UCL Institute of Neurology. Derived data supporting the findings of this study are available from the corresponding author.

## Results

We assessed 25 players diagnosed with an mTBI during a match (Table 1). For the 25 mTBI players, 36 samples were obtained at three different time points, according to player availability and consent: 1 h (*n* = 14), 3–10 days (*n* = 17) and >10 days (*n* = 5) (Supplementary Table 1). Of the 14 players who donated an mTBI sample 1 h after the injury, five had follow-up sampling at the 3- to 10-day time point. We sampled 17 post-match non-head injured control players, without mTBI 1 h after a game. There was no difference in age or years of play between the players with mTBI and the non-mTBI post-match controls (*P* = > 0.7 for all) and no significant difference in the baseline biomarker levels between those who went on to have an mTBI and those who did not (Supplementary Fig. 1).

**Table 1:** Clinical features of mTBI events sampled

Total mTBI events sampled	25
Player age range (years)	20–36
Mean number (range) of reported symptoms	6 (1–22)
Mean number (range) of previous mTBIs	5 (1–12)
Signs leading to permanent removal from play: Loss of consciousness Ataxia	2/25 1/25
Musculoskeletal injuries	1/25
Mean (range) symptomatic days	2 (1–7)
Mean (range) duration of graded return to play (days)	7 (6–12)

### Group-based blood biomarker analysis

**Figure 1:**
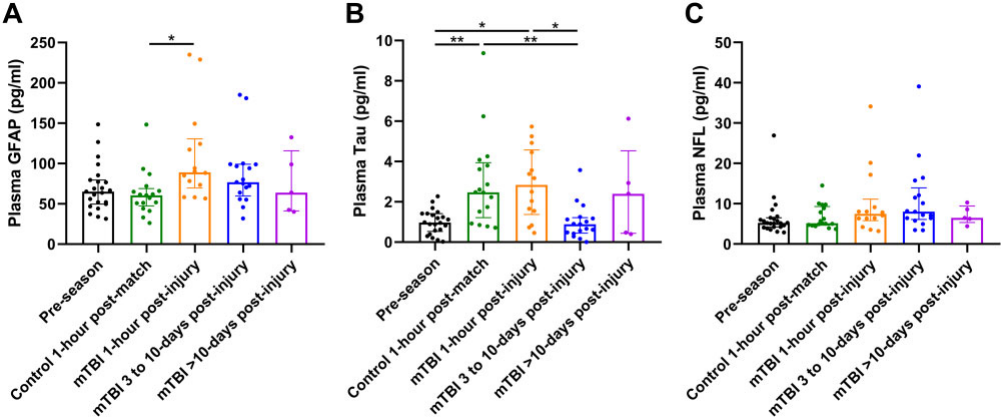
**Plasma concentration of biomarkers among different groups.** (**A**) GFAP, (**B**) tau and (**C**) NFL. Scattered dots represent the concentration for each individual. Box and bars represent the median and the interquartile range of each group. Kruskal–Wallis test with *post hoc* Dunn’s test *P < 0.05, **P < 0.01.

We assessed the difference between biomarkers (GFAP, Tau and NFL) in different groups (Fig. 1 and Supplementary Table 1).

#### Glial fibrillary acidic protein

At the group level, the mean plasma GFAP concentration was higher in mTBI samples across all the time points (1-h to > 10-day post-injury, Fig. 1A). Multiple-group comparisons further showed that the mean plasma GFAP concentration of players subject to mTBI rapidly increased to the highest level at the 1-h post-injury time point, which is higher in comparison to the non-mTBI 1-h post-match group (*P* = 0.017) and the pre-season group (*P* = 0.110). After the initial increase, plasma GFAP decreased between 3 and 10 days and returned to pre-season levels at >10-day time point. In contrast, the GFAP level remains unchanged in the non-mTBI 1-h post-match group when compared to the pre-season samples (*P* > 0.999).

#### Tau

A 2-fold increase in the mean plasma concentration of tau was observed in rugby players both with and without mTBI following a match, in comparison to pre-season levels (*P* = 0.015 and 0.007, respectively, Fig. 1B). A notable decrease in tau levels was observed in mTBI players in the 1-h post-injury group (*P* = 0.012) after 3-day post-mTBI, demonstrating that the level of tau had returned to the pre-season level in the mTBI group 3- to 10-day post-injury group.

#### Neurofilament light

No significant difference was seen in the plasma concentration of NFL between pre-season and in non-mTBI players 1 h after a match (*P* > 0.999, Fig. 1C). In mTBI cases, the concentration of NFL tended to increase from the 1-h post-injury time point in a prolonged fashion, with the highest level observed in samples collected between 3 and 10 days after a match, although no statistical significance was detected across different time points. However, the comparison between 3- and 10-day post-mTBI group and the pre-season group yielded a result that is approximate to the threshold for statistical significance (*P* = 0.070). NFL in the mTBI cases decreased to pre-season levels in samples collected after 10 days (*P* > 0.999).

### Individual level biomarker analysis

**Figure 2:**
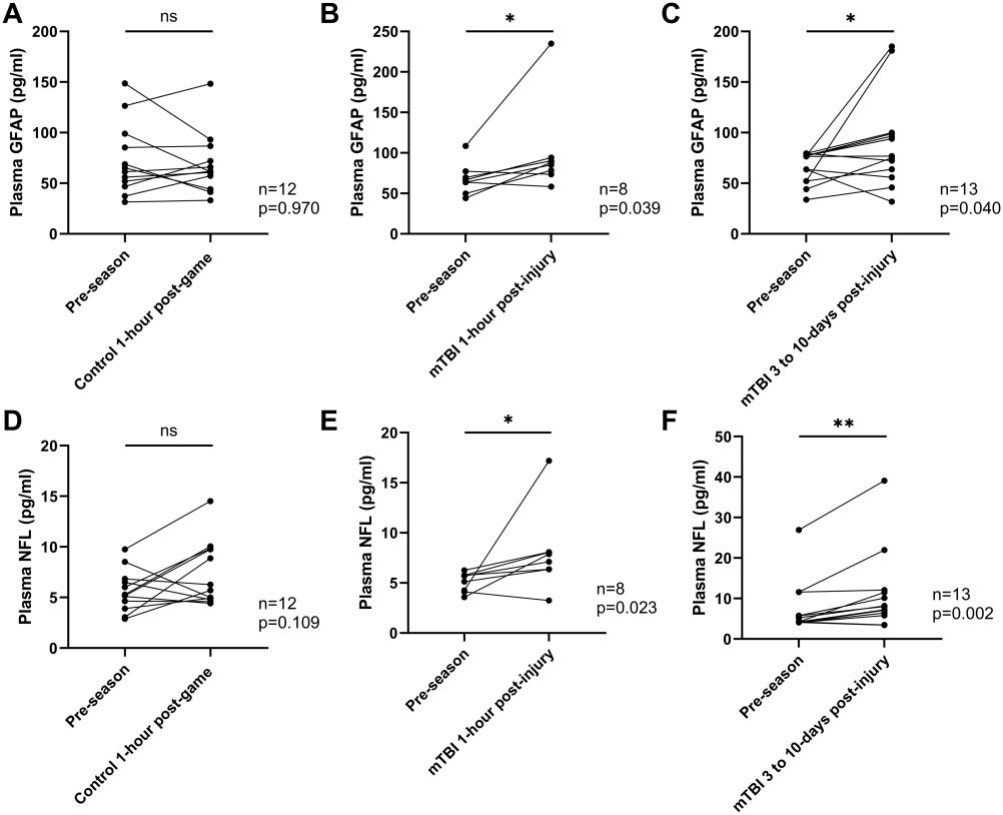
**Changes in the plasma concentration of biomarkers.** (**A**–**C**) GFAP and (**D**–**F**) NFL in the same player. The pre-season level of GFAP and NFL are compared to the level of the Control 1-h post-match group (**A** and **D**, *n* = 12), the level of mTBI 1-h post-injury group (**B** and **E**, *n* = 8) and to the level of the mTBI 3- to 10-day post-injury group (**C** and **F**, *n* = 13). Scattered dots represent the biomarker concentration for each individual; Wilcoxon matched-pairs signed rank test, *P < 0.05, **P < 0.01, ns, not statistically significant.

Wilcoxon matched-pairs signed rank test was employed to track the changes in the biomarker concentration within the same player from whom a pre-season and post-match/post-injury sample were obtained. In summary, 12 players donated both a pre-season and post-match control sample, 8 players donated both a pre-season and an mTBI 1-h post-injury sample and 13 players donated both a pre-season and an mTBI 3- to 10-day post-injury sample, the plasma concentration of GFAP was increased at the 1-h and 3- to 10-day time point when compared to the corresponding pre-season sample (*P* = 0.039 and 0.040, respectively, Fig. 2B and C). Conversely, the concentration of GFAP remained unchanged in the post-match samples from players without mTBI (*P* = 0.970, Fig. 2A).

Likewise, significant elevation in plasma NFL concentrations was seen in the comparison between samples from players with mTBI and their corresponding pre-season samples (Fig. 2E and F). Concentration of NFL was significantly higher in both the mTBI 1-h time point group and the mTBI 3- to 10-day time point group than the pre-season level (*P* = 0.023 and 0.002, respectively, Fig. 2E and F). Although an increase in NFL concentration was also observed in post-match samples from non-mTBI players, the difference was insignificant (*P* = 0.109, Fig. 2D).

**Figure 3:**
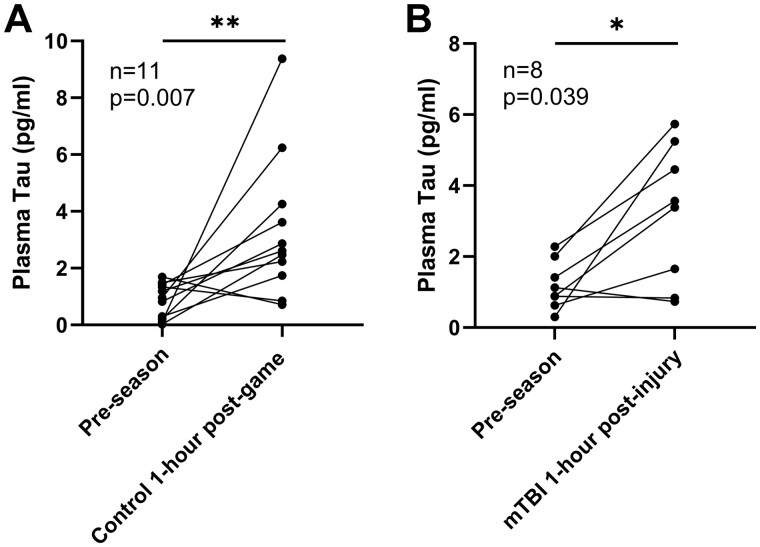
**Changes in the plasma concentration of tau within the same player.** (**A**) Pre-season level to post-match level in control group (*n* = 11) and (**B**) in the mTBI group (*n* = 8). Scattered dots represent the concentration for each individual; Wilcoxon matched-pairs signed rank test. *P < 0.05, **P < 0.01.

In contrast to GFAP and NFL, dramatic increase in the concentration of tau in samples taken at the 1-h time point was observed in both the mTBI group and the non-mTBI group (*P* = 0.007 and 0.039, respectively, Fig. 3). The difference between the mTBI 3- and 10-day post-injury group and the pre-season level is slight (*P* = 0.636, Supplementary Fig. 2), indicating a decline of plasma tau level in the 3- to 10-day post-injury group compared to the pre-season level.

As summarized in Table 2, we identified six players with particularly elevated levels of either GFAP or NFL (Players A–F) in the mTBI group (indicated in bold, Table 2). These samples displayed a higher concentration of plasma GFAP and/or NFL levels in relation to their corresponding group (identified by the interquartile method for outlier detection i.e. >1.5 interquartile ranges). Of note, Players C and D had higher levels of GFAP, but not NFL, in relation to other players with mTBI, at 3–10 days following injury. Player C had ataxia and D had transient loss of consciousness and therefore both met criteria for permanent removal from play, indicating a more severe injury. Furthermore, player C reported the highest number of symptoms (22/22) in the mTBI group and had the longest duration on return to play protocol (12 days). Players E and F both had elevated NFL levels at 3- to 10-day post-injury, with player F reporting a high number of symptoms, 16/22 (mean = 6, range: 1–22), within an hour of injury. In contrast, Players A, B and E had no clinical features to indicate more severe injury.

**Table 2:** mTBI cases with outlying elevations of NFL and GFAP

		GFAP (pg/ml)	NFL (pg/ml)
Concentration of GFAP and NFL in mTBI groups shown in Fig. 2		
mTBI 1 h	Mean (SD)	95.58 (17.96)	8.15 (1.26)
	Q3 + 1.5 ×IQR	134.8	11.53
mTBI 3–10 days	Mean	90.50 (12.78)	11.12 (2.68)
Outliers summary	Q3 + 1.5 ×IQR	149.92	19.66
Group	Player code	GFAP (pg/ml)	NFL (pg/ml)
			
mTBI 1 h	A	**234.97**	8.08
	B	88.15	**17.19**
mTBI 3–10 days	C	**185.15**	5.80
	D	**180.98**	12.09
	E	63.82	**21.98**
	F	45.80	**39.11**

IQR, interquartile range; SD, standard deviation; Q3, upper quartile.

### Diagnostic accuracy of NFL and GFAP in rugby players

**Figure 4:**
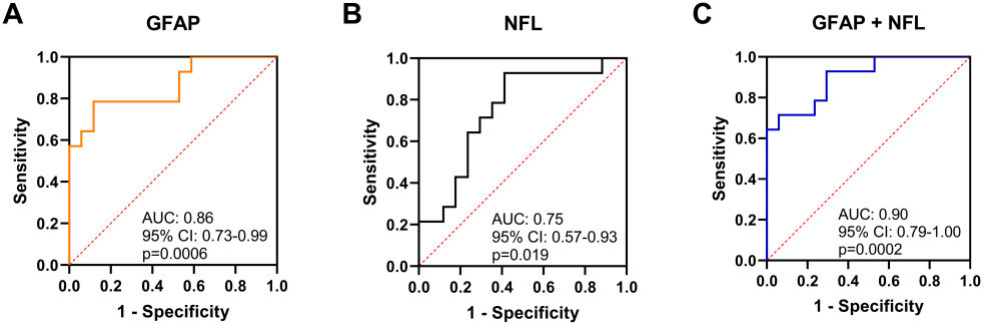
**Area under the receiver operating characteristic curve (area under curve).** (**A**) When using GFAP, (**B**) NFL and (**C**) GFAP in combination with NFL as the biomarker in differentiating mTBI 1-h post-injury group (*n* = 14) from control players 1 h after match (*n* = 17). Line of identity is indicated as dashed red line.

As GFAP and NFL were associated with rugby-related mTBI, we performed ROC analysis to test the power of these two biomarkers in separating players with mTBI from non-mTBI players at the 1-h time point (Fig. 4). Plasma GFAP level at the 1-h time point yielded an area under curve of 0.86 (95% CI 0.73–0.99, *P* = 0.0006), and NFL displayed a lower differentiation power (area under curve  = 0.75, 95% CI 0.57–0.92, *P* = 0.019). Importantly, the diagnostic accuracy is further improved when GFAP and NFL are employed as a combined biomarker, increasing the area under curve value to 0.90 (95% CI 0.79–1.000, *P* = 0.0002).

## Discussion

To date, no blood biomarker is in clinical use as an independent objective tool in the assessment of mTBI. The recent licensing of the Banyan biomarkers assay of ubiquitin C-terminal hydrolase-L1 and GFAP as a biomarker was based on the ALERT-TBI study of emergency room mTBI in which CT lesions were used as the gold standard for clinically significant TBI (Bazarian *et al.*, 2018). However, CT is a relatively insensitive tool for assessing mTBI and the vast majority of sports players with mTBI will not have CT abnormalities (Douglas *et al.*, 2018). Moreover, GFAP has recently been shown as being more sensitive than a CT for diagnosing TBI in a prospective cohort study (Yue *et al.*, 2019). Usually, mTBI sustained during contact sports does not lead to an emergency room assessment and we have evaluated these blood biomarkers in a professional sports setting.

In this study, we measured plasma GFAP, NFL and tau as potential biomarkers of sports-related mTBI and assessed the diagnostic power of these biomarkers in separating rugby players with mTBI [defined with the Head Injury Assessment protocol (Raftery *et al.*, 2016) and/or Sport Concussion Assessment Tool] from those without. Between-group analysis showed that increase in the plasma concentration of the brain astrocytic marker GFAP is associated with mTBI events. Initial elevation of the GFAP can be seen as early as 1-h post-injury and remained higher than pre-season and non-mTBI controls until >10 days.

Within-player analysis demonstrated that increased plasma concentration of GFAP and NFL is specifically related to mTBI. For the same player, significant elevation in the concentration of plasma GFAP and NFL from the pre-season level is only observed in players with mTBI and not in control players, whereas a comparable increase in plasma tau concentration is observed in players both with and without an mTBI. ROC analysis showed that both GFAP and NFL can distinguish mTBI players from non-mTBI players at the 1-h time point, and a combined biomarker of GFAP and NFL was the strongest predictor with the highest accuracy.

NFL is one of four subunits in neurofilaments and is considered a sensitive and specific marker of neuroaxonal injury (Zetterberg and Blennow, 2015; Oliver *et al.*, 2016). Serum NFL levels have been found to be higher in hockey players with persistent post-mTBI symptoms compared to those with rapidly resolving symptoms in a previous study (Shahim *et al.*, 2017). In addition, serum NFL levels were shown to increase in amateur boxers after a bout, more so in boxers with a greater number of hits to the head (Shahim *et al.*, 2018). GFAP is a monomeric intermediate filament protein predominantly expressed in the brain. GFAP is concentrated in the cytoskeleton of astrocytes and is upregulated following brain injury corresponding to astrocytic activation, making it an ideal candidate marker for brain injury patients (Pekny and Pekna, 2004). Several reports have suggest that an increased blood level of GFAP is associated with severe and moderate TBI, as well as a reliable predictor of trauma patients with mTBI (Lumpkins *et al.*, 2008; Metting *et al.*, 2012; Papa *et al.*, 2012*a*, *b*; Lewis *et al.*, 2017).

Interestingly, in our study, markedly increased levels of GFAP and NFL showed potential as sensitive predictors of a significant brain injury. Of the six mTBI players (Players A–F) with markedly elevated plasma GFAP or NFL, three had clinical features suggestive of a more significant brain injury, including loss of consciousness, ataxia and high number of symptoms/long duration on return to play. Interestingly, these players had elevations of either GFAP or NFL, suggesting that these are tracking different aspects of brain injury. Further work is needed; correlating neuroimaging, neuropsychology, biomarkers and clinical outcomes to define severity and type of brain injury. The results resonate with data from the Swedish Hockey League, where changes in serum NFL were noted in a subset of players who had the most problematic mTBIs from a clinical standpoint (i.e. with relatively prolonged mTBI symptoms), whilst most players with mTBI showed modest changes (Shahim *et al.*, 2017).

Our findings suggest that previous reports of increase in tau following sports-related mTBI may relate to peripheral trauma. T-tau has been reported to be significantly increased post-mTBI compared to pre-season baseline in ice hockey players (Shahim *et al.*, 2014). Plasma levels peaked immediately post-mTBI and decreased during rehabilitation; 1-h post-mTBI correlated with the number of days it took for symptoms to resolve. Gill *et al.* (2017) similarly found that higher levels of plasma tau, measured within 6 h of mTBI, significantly related to prolonged symptoms. However in our cohort, plasma tau was also significantly raised in players both with and without mTBI events 1 h after the match. As with previous studies on tau, this may reflect increased neuronal activity induced by physical exercise or peripheral nerve injury as opposed to acute brain axonal injury (Knaepen *et al.*, 2010). Peripheral nerve contains an extended tau (‘big tau’) comprising 695 amino acids (Goedert *et al.*, 1992; Boyne *et al.*, 1995). Current immunoassays are unable to distinguish central nervous system tau from peripheral nervous system tau, hence in our study it is unknown whether, or by how much, plasma tau is contributed to by the release of peripheral tau after brain injury. This finding highlights the need for caution in using tau as a marker of mTBI in sport, using current assays.

The main limitations of this study are the limited sample size and lack of sampling at all time points for all players, an inherent challenge in a cohort of professional athletes, which affects the full assessment of individual players prior to and following an mTBI and hence the reliance upon group comparisons. Furthermore, we have studied blood-based biomarkers, which may not reflect central nervous system changes as accurately as CSF would; transit through the blood brain barrier and enzymatic alterations of biomarkers can affect blood concentrations (Kawata *et al.*, 2016; Wright *et al.*, 2016). Moreover, the suboptimal nature of clinical mTBI diagnostics, which is based on consensus guidelines (i.e. the lack of a gold standard) is also an important limitation.

We have shown that blood biomarkers reflecting brain injury are deranged following head injury in rugby matches. The brain astrocytic marker GFAP is almost doubled in blood 1 h following a rugby-related mTBI. NFL and GFAP have potential in discriminating sports-related mTBI from players without an mTBI. Further studies on a larger cohort, including associations with clinical features, may increase the reliability of these neuronal and glial biomarkers as objective tools in the evaluation of mTBI.

## Supplementary Material

fcaa137_Supplementary_Data

## Abbreviations

GFAP =: glial fibrillary acidic protein
mTBI =: mild traumatic brain injury
NFL =: neurofilament light
UCL =: University College London
