# Supplementary material for: Plasma glial fibrillary acidic protein and neurofilament light chain, but not tau, are biomarkers of sports-related mild traumatic brain injury
Source: Brain Commun. 2020 Sep 7;2(2):fcaa137. doi: 10.1093/braincomms/fcaa137 (PMC7846133; doi:10.1093/braincomms/fcaa137)
Supplement: fcaa137_Supplementary_Data [file fcaa137_supplementary_data.zip › Supplementary Figures and legend.pdf]

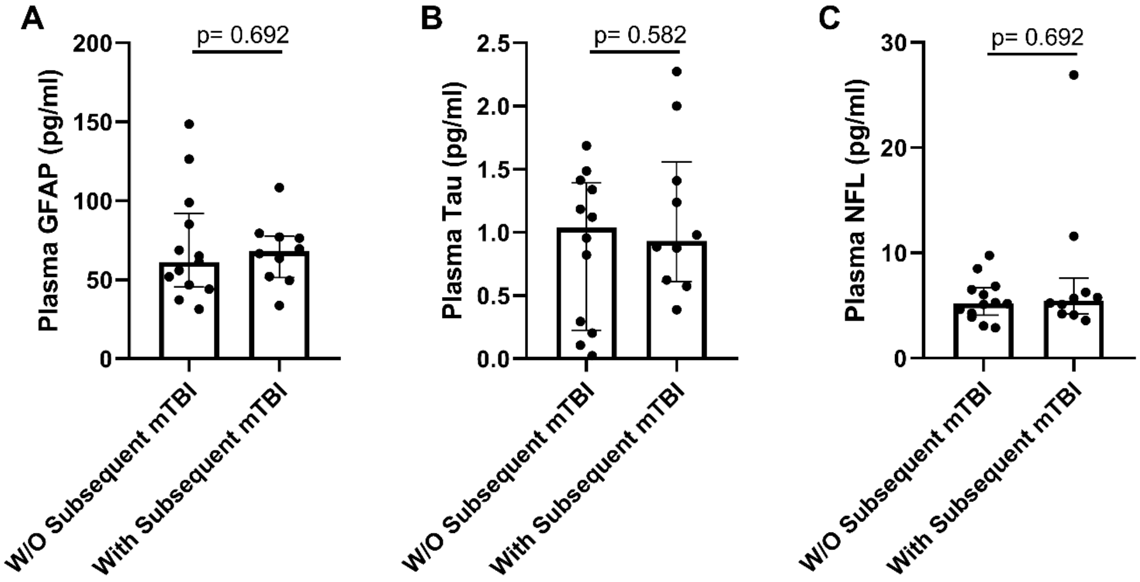

Supplementary Figure 1. Comparison of plasma level of GFAP (A), Tau (B) and NFL (C) within the pre-season group, between players not subject to subsequent mTBI events in a match (W/O Subsequent mTBI, n=13) and the players subsequently subject to mTBI (With subsequent mTBI, n=10). GFAP (A) Box and bars represent the median and the interquartile range of each group. Mann-Whitney test.

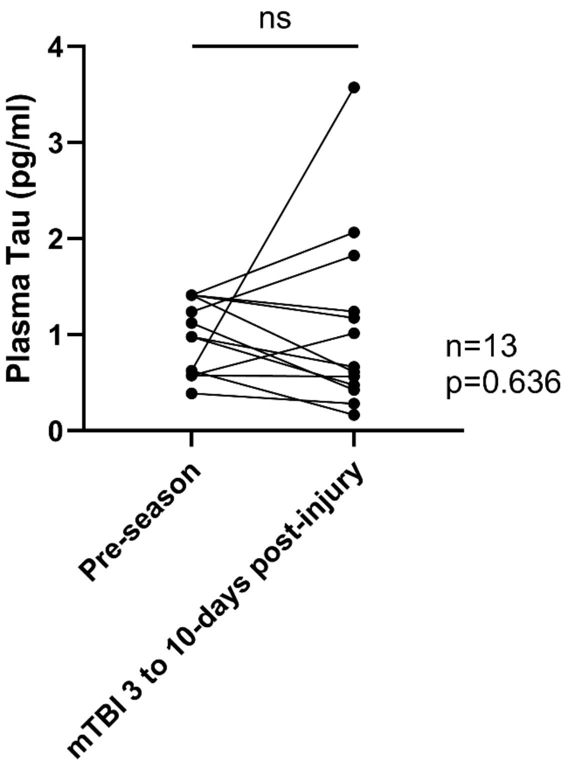

Supplementary Figure 2. Changes in the plasma concentration of tau within the same player from pre-season level to the mTBI 3 to 10-days level. Scatted dots represent the concentration of plasma tau for each individual; Wilcoxon matched-pairs signed rank test.
