# Supplementary material for: Plasma glial fibrillary acidic protein and neurofilament light chain, but not tau, are biomarkers of sports-related mild traumatic brain injury
Source: Brain Commun. 2020 Sep 7;2(2):fcaa137. doi: 10.1093/braincomms/fcaa137 (PMC7846133; doi:10.1093/braincomms/fcaa137)
Supplement: fcaa137_Supplementary_Data [file fcaa137_supplementary_data.zip › Supplementary Table 1.docx]

**Supplementary Table 1**

**Plasma biomarker concentrations in pre-season, mTBI and post-match controls**

|  | **Pre-season**  **(n= 23)** | **Control 1-hour post-match (n=17)** | **mTBI 1-hour post-injury**  **(n=14)** | **mTBI 3 to 10 days post-injury (n=17)** | **mTBI >10 days post-injury (n=5)** |
| --- | --- | --- | --- | --- | --- |
| **Age, mean (SD)** | 25.2 (3.97) | 25.1 (4.27) | 26.7(4.53) | 26.5 (2.87) | 26.4 (3.23) |
| **Years of professional play,**  **mean (SD)** | 7.83 (2.89) | 7.00 (3.54) | 7.50 (3.76) | 7.71 (2.02) | 7.40 (3.65) |
| **Previous mTBI (SD)** | 2.61 (2.59) | 1.47 (1.38) | 4.00 (3.14) | 5.41 (4.14) ^a^ | 5.20 (4.60) |
| **Biomarker Concentration median (IQR)** | | | | | |
| **GFAP** | 65.12  (49.64-79.48) | 60.61  (47.45-68.94) | 89.11 ^b^  (69.66-130.5) | 76.71  (59.79-98.96) | 63.91  (41.21-115.6) |
| **Tau** | 0.97*  (0.53-1.41) | 2.47 ^c^  (1.21-3.94) | 2.84 ^d^  (1.37-4.57) | 0.89 ^e,f^  (0.45-1.21) | 2.39  (0.43-4.53) |
| **NFL** | 5.23 ^#^  (4.22-6.51) | 5.15^#^  (4.70-9.32) | 7.49 ^#^  (5.83-11.15) | 8.01 ^#^  (6.11-13.93) | 6.46 ^#^  (5.350-9.452) |
| **Biomarker Concentration (mean ± SD)** | | | | | |
| **GFAP** | 69.49 ± 29.1 | 62.94 ± 27.8 | 109.7 ± 58.1 | 87.1 ± 41.2 | 75.5 ± 39.6 |
| **Tau** | 0.99 ± 0.6 | 2.96 ± 2.2 | 2.87 ± 1.8 | 0.99 ± 0.9 | 2.46 ± 2.3 |
| **NFL** | 6.54 ± 4.9 | 6.74 ± 3.0 | 10.13 ± 8.4 | 11.07 ± 8.8 | 7.21 ± 2.2 |
| **^a^** mTBI 3 to 10-days vs. Control 1-hour, p=0.003 < 0.01  ^b^ mTBI 1h vs. control 1-hour, p=0.017 < 0.05  ^c^ Control 1-hour vs. pre-season, p=0.007 < 0.01  ^d^ mTBI 1-hour vs. pre-season, p=0.015 < 0.05  ^e^ mTBI 3-10 days vs. Control 1-hour, p=0.005< 0.01  ^f^ mTBI 3-10 days vs. mTBI 1 hour, p=0.012 < 0.05  ^#^ no differences have reached statistical significance in this study  *one sample failed the measurement of tau | | | | | |
